# Supplementary figures and images for: Human-like NSG mouse glycoproteins sialylation pattern changes the phenotype of human lymphocytes and sensitivity to HIV-1 infection
Source: BMC Immunol. 2019 Jan 7;20:2. doi: 10.1186/s12865-018-0279-3 (PMC6322283; doi:10.1186/s12865-018-0279-3)

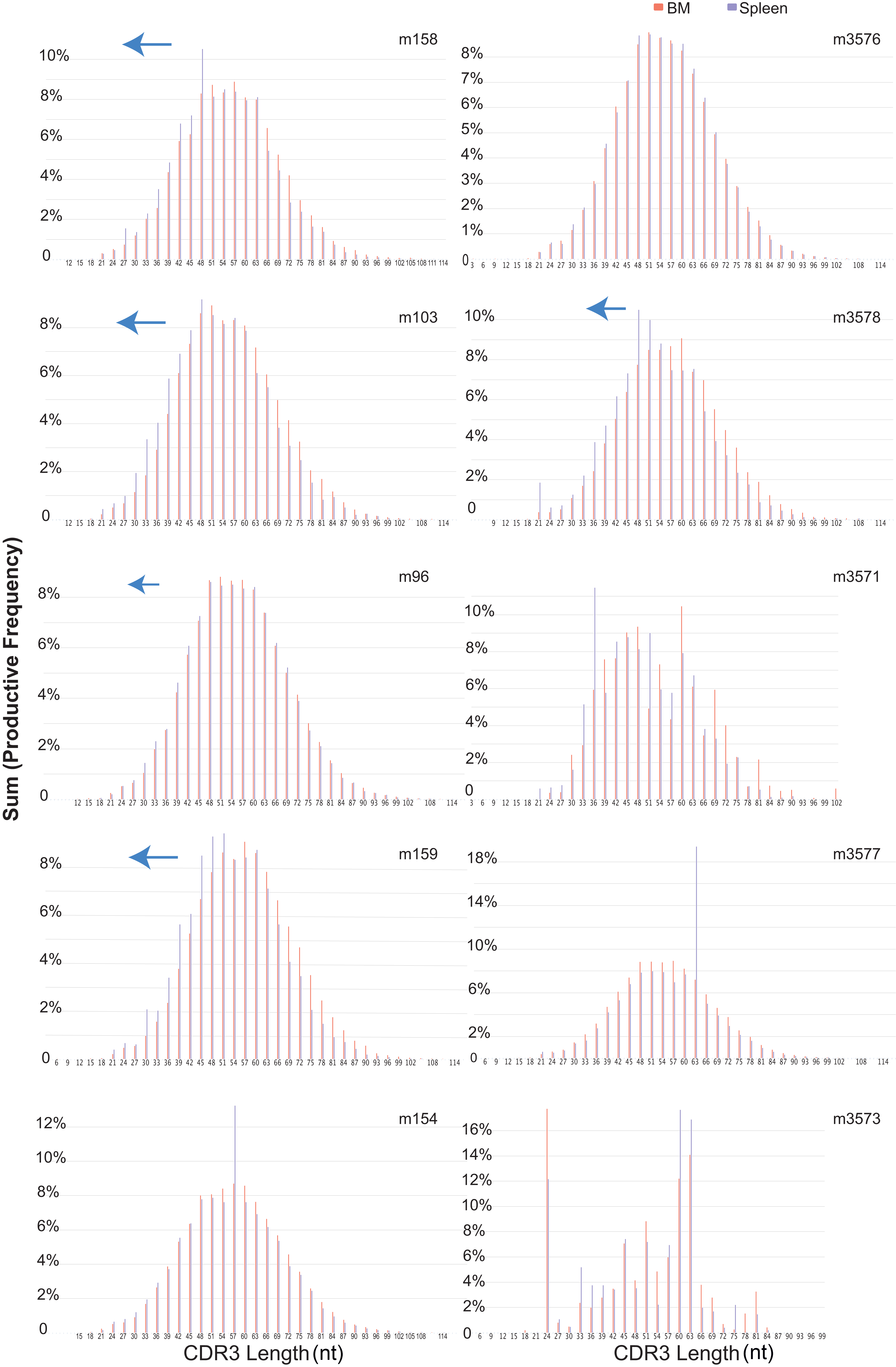

Supplement: Supplementary file 2 — Figure S2. IgH CDR3 length in bone marrow and spleen in NSG-cmah−/− (m158, 103, 96, 159, 154) and NSG wild type mice (M376, 3578, 3571, 3577, 3573). Arrows indicate shortening of CDR3 length in spleen (orange bars) compared to the bone marrow (blue bars) samples in the same animal. (PDF 1537 kb) [file 12865_2018_279_MOESM2_ESM.tif]

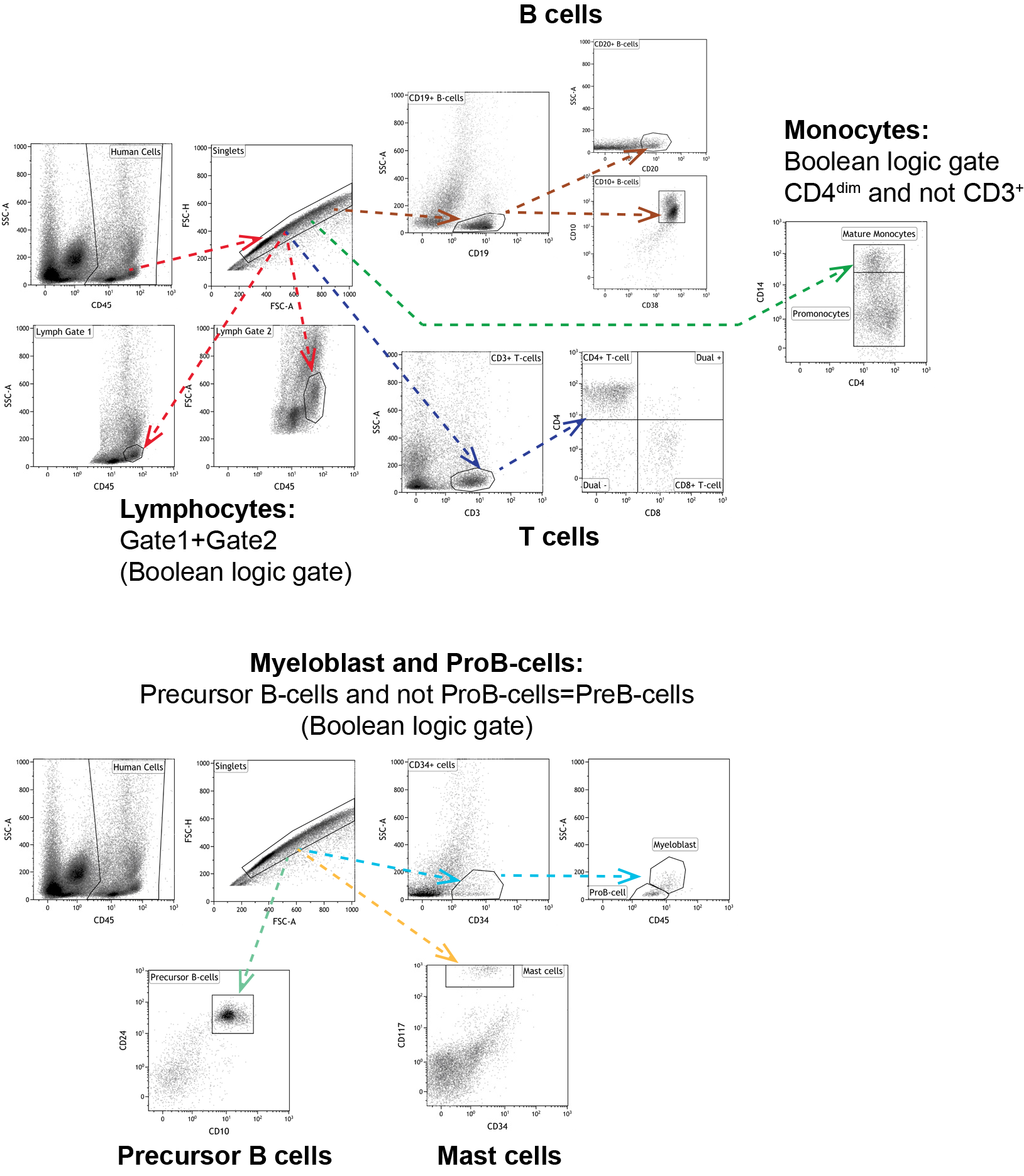

Supplement: Supplementary file 6 — Figure S6. Humanized bone marrow gating strategies. The percentage of lymphocytes was enumerated based on two CD45 by light scatter displays so that cells had to be present in both gates to be considered lymphocytes. T-cells, NK-cells, and monocytes were enumerated using a T/NK-cell cocktail containing CD3, CD4, CD7, CD8, CD14, CD16, and CD56. T-cells were identified as CD3+, low side light scatter events and were further characterized for CD4 and CD8 expression. NK-cells were isolated using a low side light scatter (SS) gate on the CD45 by side light scatter histogram. The low SS cells were characterized for CD56 and CD16 expression to enumerate the two NK-cell subsets (not shown here). Monocytes were isolated using a Boolean logic gate as CD4dim and CD3neg cells. Promonocytes and mature monocytes were identified based on CD14 expression density. CD19+, CD10+, and CD20+ B-cells were enumerated using a B-cell specific cocktail containing kappa, lambda, CD10, CD19, CD20, CD24, and CD38. CD19-positive, low SS cells were gated to enumerate total B-cells and precursors. The CD19-positive B-cells were characterized for expression of CD10 to identify the B-cell precursors and CD20 to identify transitional to mature B-cells. Myeloblasts, proB-cells, preB-cells, mast cells, and granulocytes were enumerated using a myeloid cell cocktail containing HLA-DR, CD10, CD13, CD24, CD33, CD34, and CD117. CD34-positive events were gated on a CD34 by SS histogram and were characterized as myeloblasts or pro B-cells based on the CD45 by SS profile. Total B-cell precursors were isolated based on HLA-DR, CD10, and CD24 co-expression. PreB-cells were calculated using Boolean logic as total B-cell precursors and not proB-cells. Mast cells were enumerated as CD117bright events on a CD34-positive by CD117 display. Finally, the granulocytes were estimated based on a CD45 by high SS gate that excluded the CD117bright mast cells. (PDF 1680 kb) [file 12865_2018_279_MOESM6_ESM.tif]

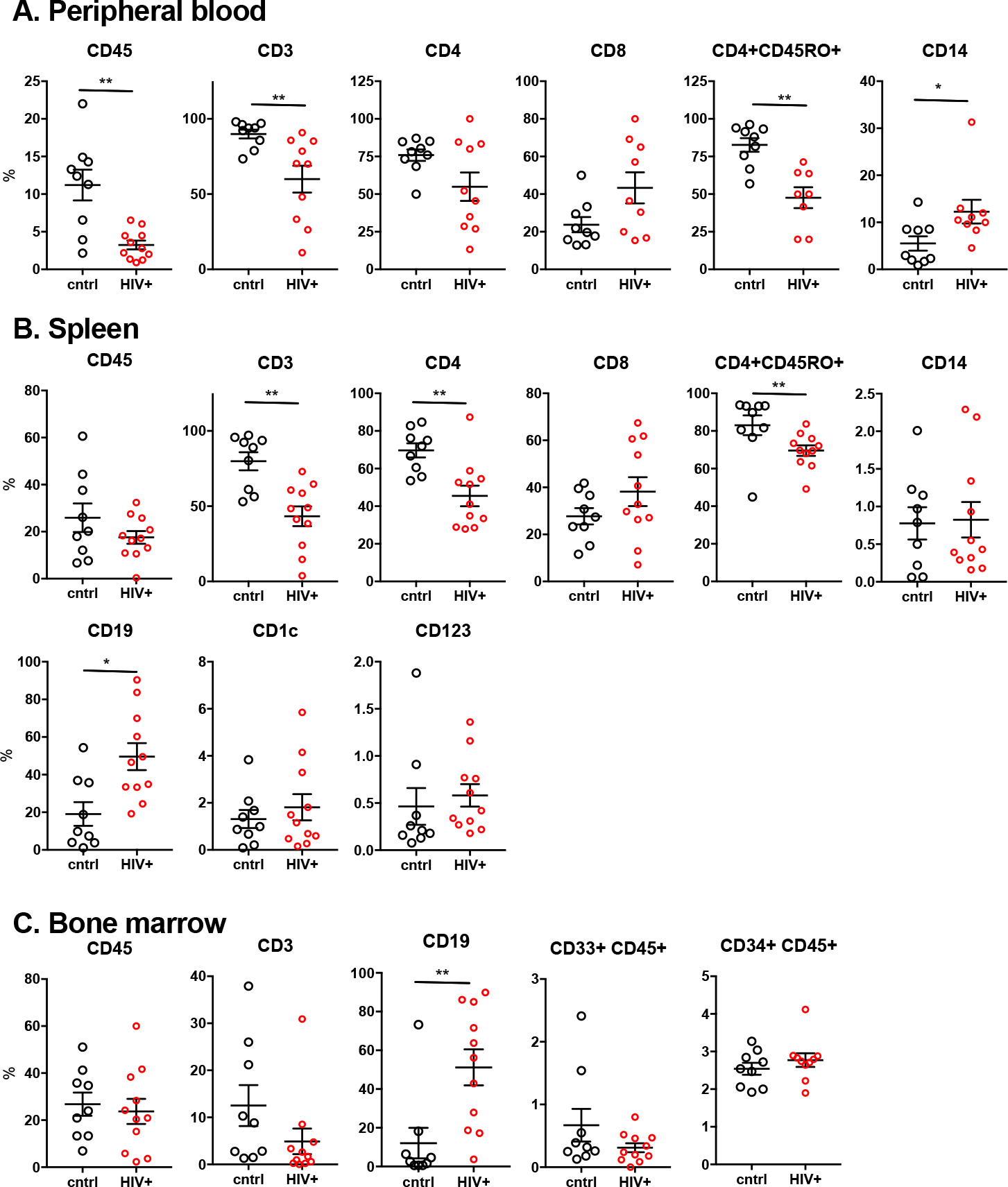

Supplement: Supplementary file 7 — Figure S7. Profile of human cells in NSG-cmah−/− at 9 weeks post HIV-1 infection. NSG-cmah−/− mice were infected with HIV-1ADA intraperitoneally at 5 months of age. At 9 weeks post-infection, samples were collected for FACS analyses of the peripheral blood, spleen, and bone marrow. A, Human cell profile in peripheral blood. FACS gating strategies used: human CD45/CD3/CD14; CD3/CD4/CD8; CD4/CD45RO. B, For spleen additional analysis included: human CD45/CD14/CD123/CD1c and CD45/CD19. C, Bone marrow analyses was done for CD45/CD3/CD19 and lineage negative CD3−/CD19− human CD34+ and CD33-positive cells. Individual mouse and means with SEM are shown. P values were determined with Mann-Whitney test. P ≤ 0.05 were considered significant. Reconstituted at variable levels, NSG-cmah−/− mice showed high sensitivity to HIV-1 infection with significantly decreased numbers of human T-cells (predominantly helper T-cells) and CD4+CD45RO+ memory T-cells in peripheral blood and spleen. In contrast, CD19-positive mature B-cells (spleen) and B-cell precursors (bone marrow) were significantly increased in NSG-cmah−/− mice following HIV exposure. Results for NSG mice at 9 weeks after HIV-1 infection not shown as only 3 animals were available for FACS analyses with significant variability. (PDF 951 kb) [file 12865_2018_279_MOESM7_ESM.tif]
